# Combination treatment with synthetic gRNA/Cas12a and gRNA/Cas9 ribonucleoproteins disrupts HIV replication and expression

**DOI:** 10.1016/j.isci.2026.115606

**Published:** 2026-04-03

**Authors:** Puja Banik, Ling Wang, Madison Schank, Jaeden S. Pyburn, Addison C. Hill, Yi Zhang, Juan Zhao, Holly K. Orfield, Tabitha O. Leshaodo, Janet W. Lightner, Xiao Y. Wu, Shunbin Ning, Mohamed El Gazzar, Jonathan P. Moorman, Zhi Q. Yao

**Affiliations:** 1 Center of Excellence in Inflammation, Infectious Disease and Immunity, Quillen College of Medicine, East Tennessee State University, Johnson City, TN 37614, USA; 2 Department of Internal Medicine, Division of Infectious, Inflammatory and Immunologic Diseases, Quillen College of Medicine, ETSU, Johnson City, TN 37614, USA; 3 HCV/HBV/HIV Program, James H. Quillen VA Medical Center, Department of Veterans Affairs, Johnson City, TN 37614, USA

**Keywords:** Genetic engineering, Immunology, Virology

## Abstract

Eradication of HIV is challenging because of the integration of proviral DNA in reservoir cells. In this study, we evaluated the antiviral effects of synthetic gRNA/Cas12a and gRNA/Cas9 ribonucleoproteins (RNPs) in HIV-infected T cells and demonstrated their specificity and efficacy in disrupting HIV gene replication and expression. Notably, sequential or combinatorial treatments with gRNA4/Cas9 and/or gRNA5/Cas12a RNPs elicited the most effective antiviral effect, with significant reduction in integrated proviral DNA, HIV mRNA, early and late reverse transcripts, and p24 protein levels. DNA sequencing revealed a high rate of insertion and deletion or knockout frequencies at the HIV target genes. Gene alignment analysis showed a high level of conservation with both gRNA4/Cas9 and gRNA5/Cas12a target sequences across diverse regional HIV strains, indicating their potential to target different HIV strains across the world. This study indicates that synthetic gRNA4/Cas9 and gRNA5/Cas12a RNPs can be used for HIV gene disruption and viral eradication.

## Highlights

• Synthetic gRNA/Cas12a and gRNA/Cas9 RNPs effectively suppress HIV replication
• DNA sequencing shows high indel and KO rates at globally conserved HIV target sites
• Sequential treatment with gRNA4/Cas9 and gRNA5/Cas12a RNPs enhances HIV suppression
• Repeated combination RNP treatments represent a promising strategy for HIV eradication

## Graphical abstract

**Figure:**
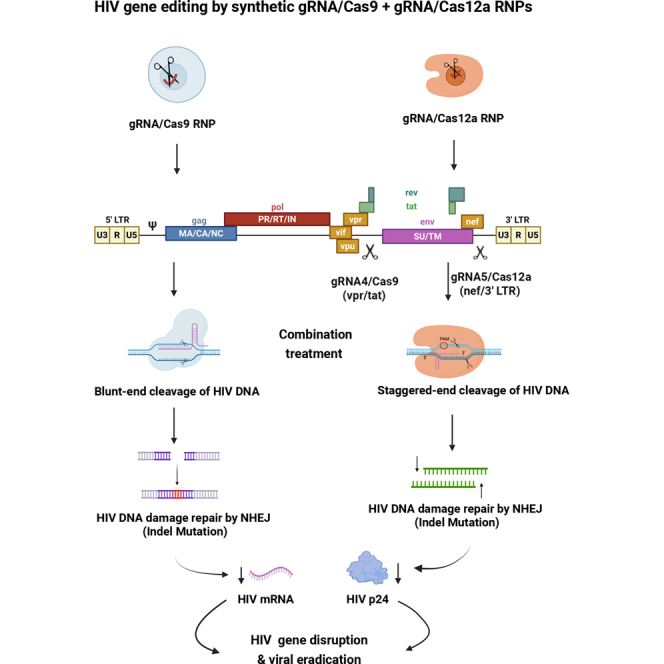

## Introduction

HIV infection is a global public health problem, and its eradication is a major challenge.^1^^,^^2^ While current antiretroviral therapy (ART), using small nucleotides to inhibit the function of HIV enzymes (such as reverse transcriptase, protease, and integrase), can effectively halt HIV replication by interfering with multiple stages in the viral life cycle, ART cannot completely eliminate the virus to eradicate HIV infection, primarily due to proviral DNA integration into the host cell genome, which remains latent but replication-competent, forming HIV reservoirs.^3^^,^^4^ These HIV reservoirs, residing mainly in resting memory CD4 T cells and/or blood/tissue monocytes/macrophages (Mo/M_Φ_), including brain microglial cells,^5^^,^^6^ are found in unique anatomical sites or sanctuaries such as lymphoid organ tissue and the central nervous system, which display poor drug accessibility and immune surveillance.^7^^,^^8^ Thus, developing potential genetic approaches to disrupt integrated proviral DNA and block HIV transcription/translation in reservoir cells is an unmet medical need for a functional and durable HIV cure.

Among the genetic approaches being utilized for targeted gene editing,^9^^,^^10^^,^^11^ the clustered regularly interspaced short palindromic repeats (CRISPR)-CRISPR-associated protein 9 (Cas9) technology is an appealing approach due to its simplicity and design flexibility.^12^^,^^13^ Previous studies have shown that CRISPR-Cas9-mediated gene editing can disrupt integrated proviral DNA and preclude HIV reactivation/replication.^14^^,^^15^^,^^16^^,^^17^^,^^18^^,^^19^^,^^20^^,^^21^^,^^22^ However, this approach faces several challenges for clinical applications, in particular, off-target effects and poor drug delivery. Therefore, selecting guide RNAs (gRNAs) targeting specific HIV genomes that are conserved among major genotypes and subtypes of HIV strains prevalent from different countries worldwide and delivering the selected therapeutics to target (reservoir) cells for on-target gene editing with minimal off-target effects are two critical steps in the development of CRISPR-Cas-based therapeutics. Additionally, the current CRISPR-Cas expression and delivery systems often require viral or non-viral vectors, which pose safety concerns for application in humans.^23^^,^^24^ As such, synthetic gRNA/Cas ribonucleoprotein (RNP) is an attractive non-viral formulation with multiple advantages, including rapid DNA cleavage, minimal off-target effects, low-risk insertional mutagenesis, ease of synthesis and gRNA multiplexing, and readiness for clinical use.^25^^,^^26^

CRISPR-Cas9-mediated gene editing leads to cleavage and disruption of the target DNA; meanwhile, the non-homologous end joining (NHEJ) pathway in host cells can partially repair the DNA damage, leading to insertion and deletion (indel) or knockout (KO) mutations and generating transcriptionally active variants that facilitate viral escape.^27^^,^^28^^,^^29^^,^^30^^,^^31^ Compared to Cas9, which often requires 2–3 gRNAs to avoid viral escape,^15^^,^^22^^,^^31^^,^^32^ Cas12a is a DNA endonuclease that is more specific and powerful in targeted gene editing.^33^^,^^34^^,^^35^ A recent study utilizing a lentiviral expression approach to compare the gene editing efficacy of CRISPR-Cas9 and CRISPR-Cas12a revealed superior antiviral activity for CRISPR-Cas12a, as it achieved complete HIV inactivation with only a single gRNA.^36^ Given their different mechanisms of action with different protospacer-adjacent motifs (PAMs) that lead to different DNA cutting sites and ends (i.e., blunt-end cleavage by Cas9 and staggered-end cleavage by Cas12a),^10^^,^^11^^,^^12^^,^^13^^,^^37^ we hypothesized that a combination treatment of HIV-infected cells with multiplexed gRNA/Cas12a and gRNA/Cas9 RNPs will lead to DNA damage at multiple sites with different cut ends and thus be more effective in disrupting proviral DNA and suppressing HIV gene transcription and translation.

We have previously reported inhibition of HIV replication using synthetic gRNA/Cas9 RNPs.^22^ In the current study, we further evaluated the antiviral effects of a combination treatment with gRNA/Cas12a and gRNA/Cas9 RNPs in latent HIV-infected T cells. Our results demonstrated that these synthetic RNPs can significantly disrupt HIV gene activities. Notably, sequential treatment with selected gRNA4/Cas9 followed by gRNA5/Cas12a, or vice versa, enhanced the antiviral activity. Repeated treatment of HIV-infected cells with a combination of gRNA4/Cas9 and gRNA5/Cas12a RNPs almost completely abolished HIV replication and expression, providing proof-of-concept that synthetic gRNA4/Cas9 plus gRNA5/Cas12a RNPs can be utilized as a prominent gene editing therapeutic to eradicate HIV infection.

## Results

### Designing gRNA/Cas12a targeting the HIV genomes

**Figure 1:**
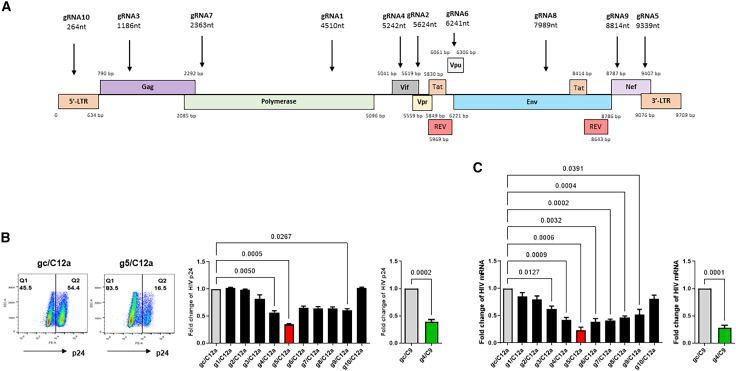
Synthetic gRNAs/Cas12a RNPs inhibit HIV replication and expression in J1.1 cells (A) The Cas12a-specific gRNAs targeting the HIV genome. The linear mapping and overlapping of non-coding and protein-coding genes within the HIV genome, as well as the specific targeting sites of ten gRNAs for Cas12a, are shown. (B) Flow cytometry analysis of HIV p24 protein expression in J1.1 T cells treated with specific RNPs. The data represent results from five independent experiments. The p24 protein expression levels in RNP-treated J1.1 T cells were normalized to the gc/C9 or gc/C12a control. (C) RT-qPCR analysis of HIV mRNA levels in J1.1 T cells treated with specific RNPs. The data are presented as the mean ± SEM of five independent experiments. Relative HIV mRNA expression levels in RNP-treated J1.1 T cells were normalized to the gc/C9 or gc/C12a control. For simplicity and clarity, the non-targeting scramble controls are labeled as gc/C9 and gc/C12a. The specific gRNA/Cas RNPs are indicated as g4/C9 for gRNA4/Cas9 and g1/C12a through g10/C12a for gRNA1-10/Cas12a (e.g., g1/C12a, g2/C12a, …, g10/C12a) throughout the figures and text. The comparison between two groups was analyzed by a paired *t* test, and comparisons among multiple groups were carried out using a parametric one-way ANOVA test, based on the normality results. *p* values <0.05 were considered statistically significant, and *p* values <0.01 were considered very significant.

To develop synthetic RNP-based therapeutics for HIV gene editing, we designed specific gRNAs for Cas12a that can cleave HIV DNA and disrupt viral replication without off-targeting the human genome. We first mapped the HIV genome using NCBI GenBank (PubMed Accession number AF324493.2, strain pNL4-3) and identified 11 potential HIV target genes, including the 5′ and 3' LTRs (long terminal repeats); three major polyproteins: Gag (group-specific antigen), Pol (polymerase), and Env (envelope); two regulatory protein: Tat (*trans*-activator of transcription) and Rev (regulator of virion expression); and four accessory proteins: Vif (virion infectivity factor), Vpu (viral protein u), Vpr (viral protein r), and Nef (negative regulatory factor). The linear mapping and overlapping of these non-coding and protein-coding genes, including their specific lengths and locations within the HIV genome, are shown in Figure 1A.

To design virus-specific gRNAs for Cas12a to target and cleave these HIV genes, we used an online CRISPR gRNA design tool (CHOPCHOP^38^) to select ten gRNAs that can target one or more HIV genes (Figure 1A). The selected gRNAs were cross-examined against the human genome to predict any off-target effects, which is a primary concern in HIV gene therapy. Therefore, we selected ten gRNAs for Cas12a based on their highest on-target (within HIV sequences that are critical for viral replication) and lowest off-target (0% overlap within the human genome) values (Table 1). These gRNAs were designed to target the HIV DNA sequences adjacent to the canonical PAM (TTTN, where N can be any nucleotide) that can be recognized by the *Lachnospiraceae bacterium (L.b*. *ND2006)-derived* Cas12a (i.e., Cpf1). Each gRNA consisted of a 21-nucleotide (nt) scaffolding region (TAATTTCTACTAAGTGTAGAT) at the 5′ end, which forms a loop structure conserved handle^39^ that is essential for the activity of Cas12a, followed by a 24-nt virus-specific region. The features of these selected gRNAs for Cas12a, including the name of the target gene(s), the start nt location, the sequence of the PAM motif (TTTN, where N is variable), the 5′-conserved handle, the target sequence, the plus (+)/minus (−) strand orientation, the percentage (%) of GC content, the self-complementarity,^40^ the predicted efficiency score,^41^ and the number of potential off-target transcripts with 0, 1, 2, or 3 mismatch (MM) possibilities, are shown in Table 1.

**Table 1:** The features of Cas12a-gRNAs targeting HIV-1 genome (pNL4-3) without off-targeting human genome

gRNA#	Target	Genomic location	PAM	5′ Conserved handle	Target sequence	Strand	GC content (%)	Self-complementarity	Efficiency score	MM0	MM1	MM2	MM3
1	Pol	seq:4510	TTTA	TAATTTCTACT AAGTGTAGAT	AGAGGAAGTAT GCTGTTTCTTGCC	–	46	0	80	0	0	0	0
2	Vpr	seq:5624	TTTA	TAATTTCTACT AAGTGTAGAT	GAGGAACTTAA GAGTGAAGCTGTT	+	42	0	74	0	0	0	0
3	Gag	seq:1186	TTTG	TAATTTCTACT AAGTGTAGAT	CCCCTGGAGG TTCTGCACTATAGG	–	58	0	70	0	0	0	0
4	Vif	seq:5242	TTTC	TAATTTCTACT AAGTGTAGAT	TCCTGTATGCA GACCCCAATATGT	–	46	0	67	0	0	0	0
5	3′ LTR/Nef	seq:9339	TTTG	TAATTTCTACT AAGTGTAGAT	ACAGCCGCCT AGCATTTCATCACG	+	54	0	63	0	0	0	0
6	Vpu/Env/Rev/Tat	seq:6241	TTTC	TAATTTCTACT AAGTGTAGAT	CACCCCCATCTCCA CAAGTGCTGA	–	58	0	59	0	0	0	0
7	Pol	seq:2363	TTTG	TAATTTCTACT AAGTGTAGAT	CCAGGAAGATGGA AACCAAAAATG	+	42	0	83	0	0	0	0
8	Tat/Env/Rev	seq:7989	TTTC	TAATTTCTACT AAGTGTAGAT	CAGAGCAACCCCA AATCCCCAGGA	–	58	0	76	0	0	0	0
9	Nef	seq:8814	TTTC	TAATTTCTACT AAGTGTAGAT	CCTTACAGCAGGC CATCCAATCAC	–	54	0	66	0	0	0	0
10	5′ LTR	seq:264	TTTG	TAATTTCTACT AAGTGTAGAT	ACAGCCTCCTAGCA TTTCGTCACA	+	50	0	63	0	0	0	0

MM0, MM1, MM2, MM3 are denoted as the number of off-target transcripts with 0, 1, 2, or 3 mismatch possibilities outside of the target genome.

Using the CHOPCHOP gRNA design tool, we predicted the number of potential off-target transcripts with 0, 1, 2, or 3 MM possibilities, which shows 0 potential off-target transcript for MM0, MM1, MM2, and MM3 (Table 1). Because of the evidence that the specificity of the targeted sequence is important for CRISPR-Cas9-mediated gene editing, we also used the Cas-OFFinder tool to predict any potential off-targets within the human genome for gRNA4/Cas9 and gRNA5/Cas12a. We observed three predicted off-target transcripts with MM3 for gRNA4/Cas9, and five off-target transcripts with MM5 for gRNA5/Cas12a, respectively. The details of these predicted off-targets within the human genome, analyzed by CasOFFinder, are shown in Tables 2 and 3.

**Table 2:** Details of predicted gRNA4/Cas9 off targets with human genome by CasOFFinder (MM3)

Off-Target #	Target	Chromosome	Position	Direction	Mismatches
1	crRNA: GGCTCTAGTCTAGGATCTACNGG	chr17	77727058	–	3
	DNA: GGtTCcAGTCTAGGATCTAgAGG				
2	crRNA: GGCTCTAGTCTAGGATCTACNGG	chr9	75200011	–	3
	DNA: GGCTCTAGaCTAaGATCTtCAGG				
3	crRNA: GGCTCTAGTCTAGGATCTACNGG	chr11	92335723	+	3
	DNA: GGCcCTAGTCTAGGATCaAgGGG

**Table 3:** Details of predicted gRNA5/Cas12a off targets with human genome by CasOFFinder (MM5)

Off-Target #	Target	Chromosome	Position	Direction	Mismatches
1	crRNA: TTTNACAGCCGCCTAGCATTTCATCACG	chr5	59993425	–	5
	DNA: TTTAACAGCCtCaTAGtATTTCATCAta				
2	crRNA: TTTNACAGCCGCCTAGCATTTCATCACG	chr1	242199856	+	5
	DNA: TTTCACAGatGaaTAGtATTTCATCACG				
3	crRNA: TTTNACAGCCGCCTAGCATTTCATCACG	chr17	11372141	+	5
	DNA: TTTAACAGCtGCaTAGtATTcCATCAtG				
4	crRNA: TTTNACAGCCGCCTAGCATTTCATCACG	chr9	81347955	+	5
	DNA: TTTAACAGCtGaaTAGCATTTCATtAtG				

### Evaluating gRNA/Cas12a RNPs for their antiviral activities

The antiviral effects of the ten selected gRNA/Cas12a RNPs were evaluated in J1.1 T cells with latent HIV infection.^22^ The gRNA/Cas12a RNP complexes were freshly assembled and then nucleofected into the cells. The gRNA control (gRNAc) for Cas12a (denoted as gc/C12a) served as the non-targeting negative control for the Cas12a system, and the gRNAc/Cas9 (gc/C9) and gRNA4/Cas9 (g4/C9) RNPs were used as negative and positive controls for the Cas9 system, respectively. Approximately 48 h after nucleofection, the cells were stimulated with PMA for 2 h (for HIV reactivation), washed twice, and incubated in PMA-free media for an additional 24 h (for latency reversal). The levels of HIV p24 protein expression in the treated cells were measured by flow cytometry. Figure 1B shows the representative pseudocolor plots and summary data of flow cytometry analysis. Among the ten gRNA/Cas12a RNPs tested, three gRNA/Cas12a RNPs (g4/C12a, g5/C12a, and g9/C12a) elicited significant suppression of HIV p24 protein expression. Notably, g5/C12a (targeting HIV Nef/3′-LTR genes) elicited the most significant (∼60%) reduction in p24 levels compared to the negative control (gc/C12a), resulting in a decrease in the p24 expression to levels similar to the positive control (g4/C9; Figure 1B, right). We also measured HIV mRNA levels in the treated cells by RT-qPCR. As shown in Figure 1C, seven gRNA/C12a RNPs (g3/C12a, g4/C12a, g5/C12a, g6/C12a, g7/C12a, g8/C12a, and g9/C12a) elicited significant reduction in HIV mRNA levels, with g5/C12a RNP showing the most significant reduction (∼70%, similar to the g4/C9 positive control shown in the right) compared to the negative control (gc/C12a, gc/C9).

Since HIV is an RNA virus with a high level of quasispecies, we asked whether our selected gRNAs could target other HIV strains. To determine this, we cross-referenced the g5/C12a targeting sequence with the National Center for Biotechnology Information (NCBI) database. We previously reported that g4/C9 targeting sequence shows high levels of conservation (>90%) across major HIV sequences from published viral strains.^22^ Similarly, the NCBI HIV database nucleotide blast revealed a total of 5,781 hits with g5/C12a target across the published HIV genomic sequences (Figure S1A). Further analysis using the clustal omega multiple sequence alignment tool revealed that the g5/C12a target site is located within a conserved sequence across HIV strains identified in different countries, including MH327753.1 isolated from Philippines, MN090715.1 from the United States, ON421508.1 from France, FJ195086.1 from Brazil, OR876504.1 from Australia, OQ979195.1 from Russia, KJ925006.1 from the United Kingdom, and EF420987.1 from China (Figure S1B).

Taken together, these results demonstrate that both g5/C12a and g4/C9 RNPs can significantly inhibit HIV gene transcription and protein expression in latent HIV-infected T cells and that these gene editing modalities have the potential to target diverse HIV strains across the world, according to the online HIV database blast and alignment analysis.

### Sequential treatment with g5/C12a and g4/C9 RNPs enhances antiviral activities

**Figure 2:**
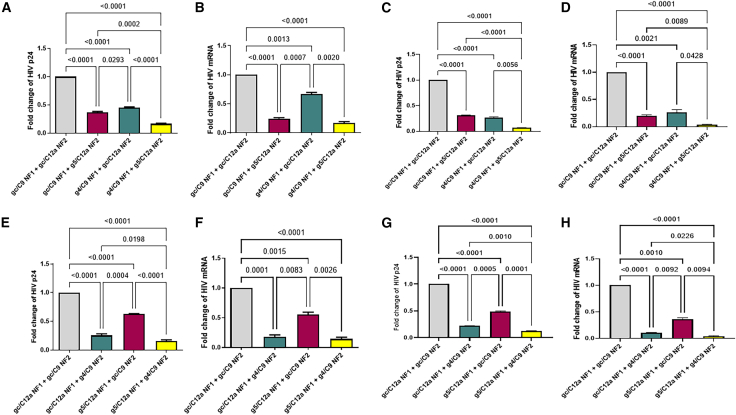
Inhibition of HIV replication and expression in J1.1 cells by sequential treatment with g4/C9 and g5/C12a RNPs (A–D) Inhibition of HIV p24 protein and mRNA expression in J1.1 T cells treated with g4/C9 nucleofection 1 (NF1) and then with g5/C12a nucleofection 2 (NF2) in the absence (A and B) and presence (C and D) of antivirals (RAL + DRV). Summary data of flow cytometry and RT-qPCR analysis from five independent experiments are shown as the mean ± SEM. Fold changes of HIV p24 protein and mRNA levels in RNP-treated J1.1 T cells were normalized to the gc/C9 + gc/C12a control. (E–H) Inhibition of HIV p24 protein and mRNA expression in J1.1 T cells treated with g5/C12a nucleofection 1 (NF1) and then with g4/C9 nucleofection 2 (NF2) in the absence (E and F) and presence (G and H) of antivirals (RAL + DRV). Summary data from flow cytometry and RT-qPCR analysis from five independent experiments are shown as the mean ± SEM. Fold changes in HIV p24 protein and mRNA levels in RNP-treated J1.1 T cells were normalized to the gc/C9 + gc/C12a control. The comparisons among multiple groups were carried out using a parametric one-way ANOVA test, based on the normality results. *p* values <0.05 were considered statistically significant, and *p* values <0.01 were considered very significant.

Given that g5/C12a and g4/C9 act through distinct mechanisms with different target sites and cleavage patterns, we investigated whether sequential treatment with g4/C9 followed by g5/C12a could enhance the overall antiviral activities. To do this, J1.1 T cells treated once (i.e., one nucleofection; NF1) were cultured for 2–3 weeks, followed by a second nucleofection (NF2). In this case, the cells treated with gc/C9 NF1 followed by gc/C12a NF2 treatment served as a negative control (shown in gray) for the gc/C9 NF1 followed by g5/C12a NF2 (i.e., g5/C12a single-targeting, shown in magenta); g4/C9 NF1 followed by gc/C12a NF2 (i.e., g4/C9 single-targeting, shown in teal); and g4/C9 NF1 followed by g5/C12a NF2 (i.e., g4/C9 + g5/C12a dual-targeting, shown in yellow) treatments. Figure 2A shows the summary data of the flow cytometry results, demonstrating that, while single-targeting treatment with either g5/C12a NF2 alone or g4/C9 NF1 alone induced significant inhibition (50%–60%) of p24 expression, g4/C9 NF1 followed by g5/C12a NF2 (i.e., dual-targeting treatment) elicited the most significant inhibition (∼75%) of HIV p24 expression compared with the control (gc/C9 NF1 + gc/C12a NF2) treatment. RT-qPCR of HIV mRNA in these cells revealed a similar inhibitory effect, with the most significant inhibition induced by the sequential dual-targeting g4/C9 NF1 + g5/C12a NF2 treatment compared with the control or single-targeting treatment (Figure 2B). Notably, a rebound in HIV expression (∼5% in p24 protein and ∼20% in HIV mRNA) was observed in the g4/C9 NF1-treated cells that were cultured for 2–3 weeks and then sequentially treated with gc/C12a NF2 (compare with the data shown in Figure 1). Since J1.1 cells have a replication-competent copy of HIV, induction of HIV by PMA stimulation will likely lead to viral spread in the culture, and this could explain the viral rebound observed with the single gRNA/Cas treatment after the reinduction of HIV in the cultured cells. To test this possibility, we performed the sequential gRNA/Cas treatment in the presence of antivirals (raltegravir and darunavir, RAL + DRV) in the culture media. While we observed patterns of HIV p24 and mRNA inhibition by the single- and dual-targeting RNP treatments similar to those without antivirals (Figures 2A and 2B), the viral rebound as well as the overall viral expression levels were reduced by the presence of the antivirals at the time of nucleofection (Figures 2C and 2D), suggesting the benefit of using both RNP and ART for durable HIV treatment.

Additionally, we examined whether sequential treatment of the g5/C12a-treated cells with g4/C9 could also improve the antiviral activity. In this case, we used the gc/C12a NF1 followed by gc/C9 NF2 treatment as a negative control (shown in gray) for the gc/C12a NF1 followed by g4/C9 NF2 (i.e., g4/C9 single-targeting, shown in teal); g5/C12a NF1 followed by gc/C9 NF2 (i.e., g5/C12a single-targeting, shown in magenta); and g5/C12a NF1 followed by g4/C9 NF2 treatments (i.e., dual targeting, shown in yellow). As shown in Figures 2E and 2F, g4/C9 NF2 treatment alone elicited a significant inhibition of HIV p24 and mRNA expression; whereas g5/C12a NF1 treatment alone resulted in a less than 50% inhibition (also a significant reduction) in HIV p24 and mRNA expression, likely due to viral rebound (∼20% bounce back in HIV p24 and mRNA compared with Figures 1A and 1B) of antiviral effect by g5/C12a NF1 treatment after 2–3 weeks of cell culture. The g5/C12a NF1, followed by g4/C9 NF2 dual-targeting treatment, showed the most significant inhibition of HIV p24 and mRNA expression compared with the control (∼80%) or single-targeting treatment. To determine whether viral spread could explain the viral rebound following the single gRNA/Cas treatment, we performed the sequential gRNA/Cas treatment in the presence of antivirals RAL plus DRV. While we observed a similar pattern of HIV p24 and mRNA inhibition by the single- and dual-targeting RNP treatments as those without antivirals (Figures 2A and 2B), the viral rebound following g5/C12a single-targeting treatment as well as the overall viral expression levels were reduced by adding antivirals at the time of nucleofection (Figures 2G and 2H), indicating a durable antiviral effect using CRISPR RNP and ART dual treatment.

Taken together, these results demonstrate that sequential treatment with synthetic g4/C9 and g5/C12a RNPs effectively enhances the antiviral activities, whereas single-targeting with g4/C9 and especially g5/C12a alone can lead to viral rebound after the NF1 treatment, which can be prevented by adding ART during the treatment to improve the overall antiviral efficacy.

### Repeated treatments with a combination of g4/C9 and g5/C12a abolish HIV replication and expression

**Figure 3:**
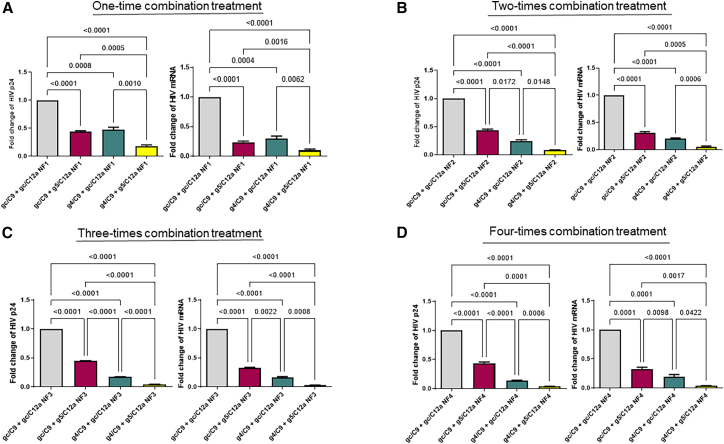
Inhibition of HIV replication and expression in J1.1 cells by repeated combination treatments with g4/C9 and g5/C12a RNPs (A) Inhibition of HIV p24 protein and mRNA expression in J1.1 T cells treated with gc/C9 + gc/C12a, gc/C9 + g5/C12a, g4/C9 + gc/C12a, or g4/C9 + g5/C12a, respectively, following one round of nucleofection (NF1). (B) Inhibition of HIV p24 protein and mRNA expression in J1.1 T cells treated with gc/C9 + gc/C12a, gc/C9 + g5/C12a, g4/C9 + gc/C12a, or g4/C9 + g5/C12a, respectively, following two rounds of nucleofection (NF2). (C) Inhibition of HIV p24 protein and mRNA expression in J1.1 T cells treated with gc/C9 + gc/C12a, gc/C9 + g5/C12a, g4/C9 + gc/C12a, or g4/C9 + g5/C12a, respectively, following three rounds of nucleofection (NF3). (D) Inhibition of HIV p24 protein and mRNA expression in J1.1 T cells treated with gc/C9 + gc/C12a, gc/C9 + g5/C12a, g4/C9 + gc/C12a, or g4/C9 + g5/C12a, respectively, following four rounds of nucleofection (NF4). All data are the mean ± SEM of five independent experiments. Fold changes of HIV p24 protein and mRNA levels in treated cells were normalized to the gc/C9 + gc/C12a control. The comparisons among multiple groups were carried out using a parametric one-way ANOVA test, based on the normality results. *p* values <0.05 were considered statistically significant, and *p* values <0.01 were considered very significant.

Transient viral inhibition by a single gRNA/Cas9-mediated HIV gene editing event can lead to viral escape over time.^27^^,^^28^^,^^29^^,^^30^^,^^31^ We and others have reported that combinatorial treatment with dual gRNA/Cas9 RNPs can improve the overall antiviral efficacy.^22^^,^^31^^,^^32^^,^^33^ Considering the limitation of transfection efficiency and different mechanisms of action in gene editing, in this study, we further determined whether repeated treatments with a combination of g4/C9 and g5/C12a RNPs can abolish viral replication and expression. In this case, J1.1 T cells were transfected with a half-dose of each RNP (which resulted in a similar inhibitory effect as the full-dose, data not shown), including gc/C9 + gc/C12a control, gc/C9 + g5/C12a, g4/C9 + gc/C12a, and g4/C9 + g5/C12a with one round of nucleofection (NF1). Approximately 48 h after treatment, the cells were stimulated with PMA for 2 h (for HIV reactivation) and incubated for an additional 24 h (for latency reversal). As shown in Figure 3A, the NF1 treatment with a combination of g4/C9 and g5/C12a RNPs resulted in a significant reduction in HIV p24 protein (∼82%) and mRNA (∼90%) levels, which is greater than the single-targeting treatment with g4/C9 or g5/C12a RNP alone. A portion of the NF1-treated cells from each group was cultured for 2–3 weeks and then subjected to a second round of nucleofection (NF2), which resulted in a substantial reduction in HIV p24 protein (92%) and mRNA (94%) levels following the combinatorial treatment with g4/C9 + g5/C12a RNPs (Figure 3B). We also nucleofected a third (NF3) and fourth (NF4) time with 2–3 weeks culture between nucleofections to determine if repeated treatments could result in additional viral suppression. As shown in Figures 3C and 3D, NF3 and NF4 treatments with combined g4/C9 + g5/C12a RNPs resulted in 98% and 99% reduction in the HIV p24 protein and mRNA levels, respectively, and almost completely abolished HIV activities. Thus, g4/C9 + g5/C12a dual-targeting treatment leads to a significant improvement over the single-targeting treatment. To observe the long-term antiviral effect of these repeated combinatorial treatments, after NF4, we cultured the cells in the presence or absence of antivirals (RAL plus DRV) for 10 weeks, followed by weekly assessment of viral rebound for 3 weeks. We did not observe any difference in the HIV p24 and mRNA levels by flow cytometry or RT-qPCR assays in J1.1 cells with or without antivirals (data not shown).

These results demonstrate that repeated combinatorial treatment with g4/C9 and g5/C12a is highly effective in suppressing HIV mRNA transcription and protein expression.

### Combination treatment with g4/C9 and/or g5/C12a reduces HIV DNA, RNA, or protein levels in HIV-infected J11.1, ACH2, and primary CD4 T cells

**Figure 4:**
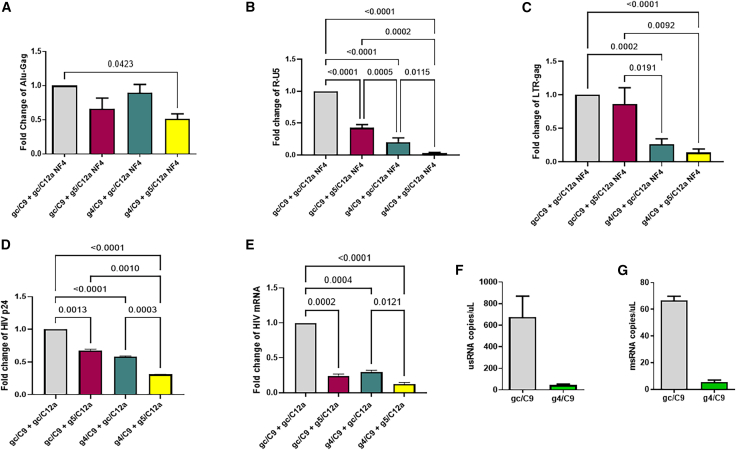
Combination treatment with g4/C9 and/or g5/C12a reduces HIV DNA, RNA, or protein levels in HIV-infected J1.1, ACH2, and primary CD4 T cells (A) Alu-gag PCR analysis of the integrated proviral DNA levels in the genomic DNA isolated from NF4-treated J1.1 T cells with NF4 treatment by gc/C9 + gc/C12a, gc/C9 + g5/C12a, g4/C9 + gc/C12a, or g4/C9 + g5/C12a RNPs, respectively. The data are the mean ± SEM of five independent experiments, normalized by β-globulin and then subtracted from both Alu and gag-only signals. (B and C) The levels of HIV early and late reverse transcripts, measured by RT-qPCR for HIV R-U5 and LTR-gag mRNAs using cDNA from J1.1 T cells following NF4 treatment with gc/C9 + gc/C12a, gc/C9 + g5/C12a, g4/C9 + gc/C12a, or g4/C9 + g5/C12a RNPs, respectively. The data are the mean ± SEM of five independent experiments. (D and E) Inhibition of HIV p24 protein and mRNA expression in ACH-2 T cells treated with gc/C9 + gc/C12a, gc/C9 + g5/C12a, g4/C9 + gc/C12a, or g4/C9 + g5/C12a, respectively, by one-time of nucleofection using the Lonza 4D-Nucleofector kit for 72 h (as described in the STAR Methods), followed by flow cytometry and RT-qPCR assays. The data are the mean ± SEM of five independent experiments. (F and G) g4/C9-KD reduces HIV usRNA and msRNA expression in CD4 T cells from viremic PLWH. Primary CD4 T cells were isolated and combined from eight viremic PLWH and then nucleofected with g4/C9 or gc/C9 RNPs. After 72 h of nucleofection, total RNAs were isolated from the treated cells; HIV usRNA and msRNA levels were quantified by ddPCR. Data are presented as copies/μL using combined CD4 T cells from eight viremic PLWH. The comparisons among multiple groups were carried out using a parametric one-way ANOVA test, based on the normality results. *p* values <0.05 were considered statistically significant, and *p* values <0.01 were considered very significant.

The integrated proviral DNA in the host cell genome is the root cause of HIV viral rebound after cessation or interruption of ART.^1^^,^^2^^,^^3^^,^^4^ The Alu-gag PCR method quantifies integrated proviral DNA by amplifying the HIV gag gene using a primer anchored in host genomic Alu repeats, thereby restricting detection of the viral genome that has undergone stable chromosomal integration. To determine the effect of this gene editing treatment on integrated proviral DNA, we assessed the levels of integrated proviral DNA after NF4 treatment using the Alu-gag PCR method.^42^ We found that g4/C9 + g5/C12a NF4 treatment significantly reduced the integrated proviral DNA in the J1.1 T cell genome (Figure 4A). To determine whether gene editing-mediated proviral DNA cleavage affects viral transcription, we measured HIV early and late RT products in these cells using RT-qPCR to amplify the R-U5 and LTR-gag transcripts,^43^ respectively. As shown in Figures 4B and 4C, the levels of early (R-U5) and late (LTR-gag) HIV RT products were significantly reduced in J1.1 T cells with NF4 treatment with the g4/C9 and/or g5/C12a RNPs, with combinatorial dual-targeting treatment exhibiting the most effective antiviral effects compared to the signal-targeting treatment.

In addition to J1.1 T cells, we also observed the inhibitory effects of the combinatorial treatment in ACH-2 T cells with latent HIV infection. As shown in Figure 4D, one-time treatment with g4/C9 and/or g5/C12a RNPs significantly reduced the levels of HIV p24 protein in ACH-2 cells stimulated with PMA (for HIV latency reversal and activation). Notably, the dual-targeting treatment with g4/C9 plus g5/C12a RNPs exhibited the most effective viral inhibition compared with g4/C9 or g5/C12a single-targeting treatment, which were all statistically significant compared with the control (gc/C9 + gc/C12a) treatment. We observed a similar level of inhibitory effects on HIV mRNA in ACH-2 cells by RT-qPCR (Figure 4E). These results indicate that g4/C9 and g5/C12a RNPs can inhibit HIV in different T cell lines with latent viral infection.

To determine whether g4/C9 knockdown (KD) can affect HIV transcription in primary CD4 T cells, we isolated CD4 T cells from eight viremic people living with HIV (PLWH, Table 4) and nucleofected the cells with g4/C9 or gc/C9 RNPs for 72 h, followed by determining HIV un-spliced RNA (usRNA) and multi-spliced RNA (msRNA) levels by droplet digital PCR (ddPCR) using specific primers (Table 5). As shown in Figures 4F and 4G, both HIV usRNA and msRNA levels were significantly reduced following g4/C9 KD compared with the control (gc/C9) treatment. This result provides proof-of-concept that this therapeutic approach is also effective in knocking down HIV in primary CD4 T cells from PLWH infected by wild-type HIV strains *ex vivo*.

**Table 4:** Characteristics of PLWH used in this study

Repository	HIV viral load	Collection date	Race	Age	Sex	ART	CD4 count
HIV-23-79	29500	3/15/2023	White	68	Male	Yes	510
HIV-23-113	29500	4/20/2023	White	68	Male	Yes	524
HIV-22-233	30	8/30/2022	White	53	Female	Yes	330
HIV-22-282	69300	10/18/2022	White	53	Female	Yes	330
HIV-22-341	505000	12/15/2022	White	45	Male	Yes	164
HIV-23-191	139000	8/24/2023	Latino	38	Male	Yes	172
HIV-23-150	46100	6/8/2023	White	33	Male	Yes	177
HIV-23-168	2480	7/13/2023	White	31	Male	Yes	249

**Table 5:** The primers and probes used for ddPCR in this study

Assay	Region	Type	Sequence (5′-3′)	Amplicon length	Reference
usRNA	Gag	Forward	5'-CATGTTTTCAGCATTATCAGAAGGA-3'	100 bp	Palmer and colleagues^53^
		Reverse	5′-TGCTTGATGTCCCCCCACT-3′		
		Probe	5′ FAM-CCACCCCACAAGATTTAAACA CCATGCTAA-Q 3'		
msRNA	tat-rev	Forward	5′-CTTAGGCATCTCCTATGGCAGGAA-3′	115 bp	Kiselinova and colleagues^54^
		Reverse	5′-GGATCTGTCTCTGTCTCTCTCTCCACC-3′		
		Probe	5′ FAM-TTCCTTCGGGCCTGTCGGGTCCC-Q 3'		
RPP30	RPP30	Forward	5′-AGATTTGGACCTGCGAGCG-3′	64 bp	Malatinkova and colleagues^55^
		Reverse	5′-GAGCGGCTGTCTCCACAAGT-3′		
		Probe	5′-FAM-TTCTGACCTGAAGGCTCTG CGCG-TAMRA-3′		

Taken together, these results demonstrate that combinatorial treatment with g4/C9 and g5/C12a is highly effective in suppressing HIV mRNA and p24 protein expression by degrading the integrated proviral DNA and inhibiting the viral reverse transcription.

### Combination treatment with g4/C9 and g5/C12a significantly reduces the production of infectious HIV particles

**Figure 5:**
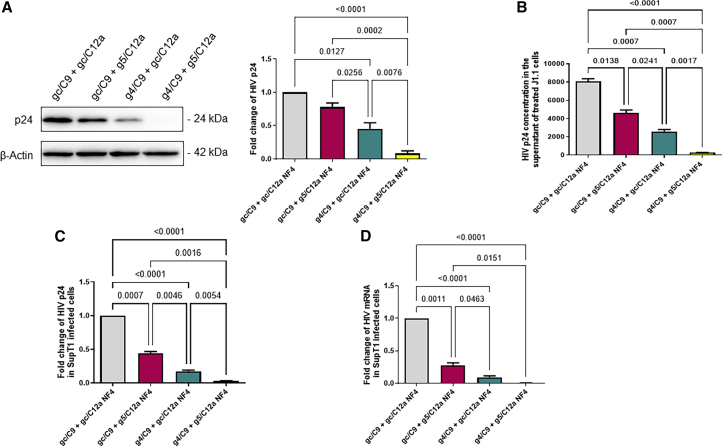
Treatment with g4/C9 and g5/C12a RNPs reduces infectious HIV particles from J1.1 cells (A) Western blot analysis of HIV p24 expression levels in J1.1 T cells following NF4 treatment with gc/C9 + gc/C12a, gc/C9 + g5/C12a, g4/C9 + gc/C12a, and g4/C9 + g5/C12a RNPs. β-actin serves as a loading control. Representative imaging and summary data from five independent experiments are shown as the mean ± SEM. (B) ELISA measuring HIV p24 protein levels in the supernatants of the J1.1 T cells following NF4 treatment with gc/C9 + gc/C12a, gc/C9 + g5/C12a, g4/C9 + gc/C12a, and g4/C9 + g5/C12a RNPs. The data are the mean ± SEM of five independent experiments. (C and D) HIV p24 (measured by flow cytometry) and mRNA levels (measured by real-time RT-qPCR) in SupT1 cells at 48 h after infection with the supernatants of J1.1 T cells following NF4 treatment with gc/C9 + gc/C12a, gc/C9 + g5/C12a, g4/C9 + gc/C12a, and g4/C9 + g5/C12a RNPs. The data are the mean ± SEM of four independent experiments. The comparisons among multiple groups were carried out using a parametric one-way ANOVA test, based on the normality results. *p* values <0.05 were considered statistically significant, and *p* values <0.01 were considered very significant.

To determine whether the CRISPR-mediated DNA cleavage reduces HIV release from J1.1 T cells, we first measured the HIV p24 protein levels in the treated cells by western blot. Figure 5A shows a representative blot (left) and densitometry data (right) revealing that the expression of HIV p24 protein was significantly suppressed in J1.1 T cells treated 4-times (NF4) with g5/C12a or g4/C9, and particularly, g4/C9 plus g5/C12a in combination. We also measured HIV p24 protein levels in the supernatants of the J1.1 T cells following NF4 treatment by ELISA. Compared to the control (gc/C9 + gc/C12a), NF4 treatment with g5/C12a, g4/C9, and especially the combination of g4/C9 plus g5/C12a RNPs, resulted in a significant reduction in HIV p24 protein levels in the culture supernatant (Figure 5B).

To further evaluate whether the treated cells produce fewer infectious viral particles, approximately 300 μL of the supernatants from J1.1 T cells with NF4 treatment were used to infect the highly permissive SupT1 cells using the spinoculation method.^22^ As shown in Figures 5C and 5D, SupT1 cells infected with supernatants from g4/C9 plus g5/C12a RNPs dual-treated cells showed the most reduced viral infection at 48 h after spinoculation, as evidenced by the significant reduction in the HIV p24 protein and mRNA levels compared to cells infected with supernatants with single RNP treatment, which was also effective in reducing HIV infection compared to the control (gc/C9 + g5/C12a)-treated cells. These data suggest that combination treatment with g4/C9 and g5/C12a effectively reduces the production of infectious HIV particles in the J1.1 T cells.

### Combination treatment with g4/C9 plus g5/C12a specifically disrupts HIV genes

CRISPR-Cas-mediated gene editing leads to targeted DNA double-strand breaks (DSBs), which are repaired by the non-homologous end joining (NHEJ) and/or homologous recombination (HR) repair pathways.^44^ The NHEJ pathway, which is the host’s primary DNA repair machinery following CRISPR-mediated gene editing and can introduce indel and KO mutations, whereas the HR pathway is high-fidelity, template-dependent DNA repair following complex DNA damages such as DNA gaps, DSBs, or DNA interstrand crosslinks.^45^^,^^46^ Thus, evaluation of indel and KO mutations in the target gene following CRISPR-Cas-mediated DNA damage and repair can determine its gene editing capability.

**Figure 6:**
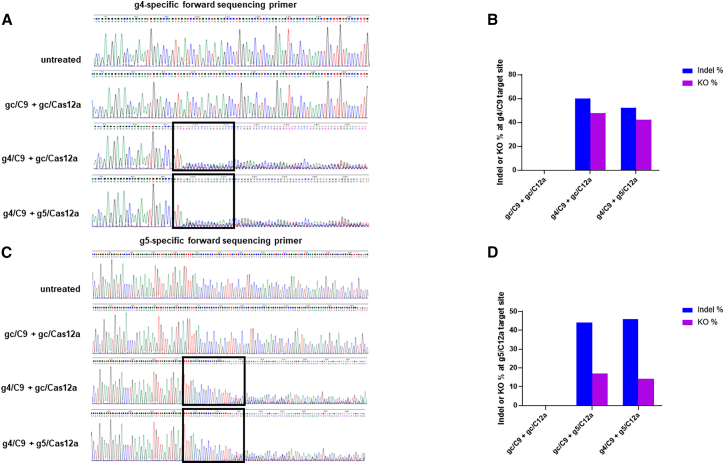
DNA sequencing reveals HIV gene disruption in J1.1 cells (A) Sanger DNA sequencing of PCR products amplified from genomic DNA isolated from J1.1 T cells without or with NF4 treatment with gc/C9 + gc/C12a, g4/C9 + gc/C12a, and g4/C9 + g5/C12a RNPs. A g4/C9 specific forward primer was used for genomic DNA amplification and DNA sequencing. The g4/C9 cleavage site is rectangle-framed. The color coding for the nucleotide bases (A, green; T, red; C, blue; and G, black) matches the color of the peaks. (B) Indels and KO frequencies at the g4 target region using a g4-forward sequencing primer to sequence the PCR-amplified DNA from cells treated with gc/C9 + gc/C12a, g4/C9 + gc/C12a, or g4/C9 + g5/C12a RNPs, respectively. (C) Sanger DNA sequencing of PCR products amplified from genomic DNA isolated from J1.1 T cells without or with NF4 treatment with gc/C9 + gc/C12a, gc/C9 + g5/C12a, and g4/C9 + g5/C12a RNPs. A g5/C12a specific forward primer was used for DNA sequencing. The g5/C12a cleavage site is rectangle-framed. (D) Indels and KO frequencies at the g5 target region using a g5-forward sequencing primer to sequence the PCR-amplified DNA fragment from cells treated with gc/C9 + gc/C12a, gc/C9 + g5/C12a, or g4/C9 + g5/C12a RNPs, respectively.

We confirmed the CRISPR-mediated HIV gene editing using genomic DNA isolated from J1.1 T cells with NF4 combination treatment by performing Sanger DNA sequencing after PCR amplification of the HIV DNA fragment using specific forward and reverse primers (Table 6). We first sequenced the DNA fragment containing the g4/C9 targeting site (HIV vpr/tat) using a g4-specific forward sequencing primer. Following a successful g4/C9-mediated DNA cleavage, DNA damage repair pathways can delete, add, or substitute random bases, introducing indel and KO mutations. These changes affect the DNA sequencing readout because the DNA analysis cannot determine the nucleotides after it reaches the g4/C9 targeting site, which often generates blunt-end DSB. As such, a CRISPR-mediated DNA cleavage could technically stop a sequence reading abruptly. As shown in Figure 6A, the sequencing readout displayed a high level of “noise” at (and beyond) the g4/C9 target site (rectangle framed) with the g4-specific forward sequencing primer in DNA from the g4/C9 or g4/C9 plus g5/C12a-treated cells, but not in DNA from the untreated and control (gc/C9 plus gc/C12a)-treated cells. Using the ClustalW pairwise alignment software, we found that the control (gc/C9 plus gc/C12a)-treated cells had 100% conservation compared to the untreated cells, while the g4/C9 and g4/C9 plus g5/C12a-treated cells had remarkable sequence mutations compared with the control-treated or untreated cells. Next, we used the Synthego Inference of CRISPR Edits (ICE) tool to analyze the indel and KO frequencies at the HIV target region (25-nucleotide upstream and 55-nucleotide downstream of the gRNA target site) via comparison with the wild-type sequence. As shown in Table 7, the ICE frequency represents the indel percentage, which includes all edit events, regardless of whether they are expected to introduce a KO. The KO-Score represents the editing events that are expected to introduce a KO (frameshift mutations or fragment deletions of 21-nucleotide or greater) and thus provides insight into how many of the contributing indels are likely to result in a functional KO of the targeted gene. As shown in Figure 6B, we found that the control (gc/C9 plus gc/C12a)-treated cells had 0% indel and KO frequencies in the g4/C9 target site. In contrast, treatment with g4/C9 or g4/C9 plus g5/C12a RNPs resulted in higher indel and KO rates (52%–60% and 42%–48%, respectively) compared with the control-treated cells.

**Table 6:** The primers used for qPCR and HIV-1 DNA sequencing

Application	Target	Forward (5′-3′)	Reverse (5′-3′)
qPCR	GAPDH	TGCACCACCAACTGCTTAGC	GGCATGGACTGTGGTCATGAG
	HIV-1 mRNA	CAGATCCTGCATATAAGCAGCTG	TTTTTTTTTTTTTTTTTTTTTTTTGAAGCAC
	β-globin	CCCTTGGACCCAGAGGTTCT	CGAGCACTTTCTTGCCATGA
	HIV-1 R-U5	GGCTAACTAGGGAACCCACTG	CTGCTAGAGATTTTCCACACTGAC
	HIV-1 LTR-gag	CAGATATCCACTGACCTTTGG	GCTTAATACTGACGCTCTCGCA
	aHuman Alu	GCCTCCAAAGTGCTGGGATTACAG	
	aHIV-1 gag		GTTCCTGCTATGTCACTTCC
Sequencing	gRNA4/Cas9	TTGGGCAGGAGTGGAAGCC	ATGAGCTCTTCGTCGCTGTCTCCGC
	gRNA5/Cas12a	CTTCCCTGATTGGCAGAACTAC	CTTGTAGCAAGCTCGATGTCAG

a The Alu-gag PCR will first amplify the Alu-gag, Alu only, and gag only, and then use the first 30-cycles of PCR products as template for the 2nd round of PCR. The integrated proviral DNA levels will be determined by the Alu-gag products subtraction of the gag only (free HIV) background signal.

**Table 7:** Indel and knock-out intensities analysis with Cas9-gRNA4 and Cas12a-gRNA5 forward primer

Label	Primer	ICE	KO-score	KI-score	ICE d	R squared	Mean discord before	Mean discord after	Guide sequences	Control sample quality score	Edit sample quality score	Indels
gc/C9+gc/C12a	g4/C9-F	0	0	None	0	1	0.0995	0.0526	GTAGATCCTAG ACTAGAGCCC	61	61	{‘0': 100.0, ‘-1': 0.0}
g4/C9 + gc/C12a	g4/C9-F	60	48	None	86	0.7	0.1557	0.6506	GTAGATCCTAG ACTAGAGCCC	61	46	{‘0': 10.0, ‘1': 17.0, ‘2': 12.0, ‘-1': 7.0, ‘-9': 12.0, ‘-20': 1.0, ‘-22': 6.0, ‘-28': 2.0, ‘-29': 2.0, ‘-30': 1.0}
g4/C9 + g5/C12a	g4/C9-F	52	42	None	83	0.69	0.1411	0.6248	GTAGATCCTAG ACTAGAGCCC	61	61	{‘0': 17.0, ‘1': 18.0, ‘2': 13.0, ‘-9': 8.0, ‘-15': 2.0, ‘-19': 1.0, ‘-22': 1.0, ‘-28': 3.0, ‘-29': 1.0, ‘-30': 3.0, ‘-35': 2.0}
gc/C9 + gc/C12a	g5/C12a-F	0	0	None	2	1	0.088	0.057	ACAGCCGCCTAG CATTTCATCACG	61	61	{‘0': 100.0, ‘-1': 0.0}
gc/C9 + g5/C12a	g5/C12a-F	44	17	None	50	0.99	0.08	0.396	ACAGCCGCCTAG CATTTCATCACG	61	61	{‘0': 55.0, ‘1': 1.0, ‘2': 3.0, ‘-1': 2.0, ‘-2': 1.0, ‘-3': 24.0, ‘-4': 1.0, ‘-5': 4.0, ‘-8': 2.0, ‘-9': 1.0, ‘10': 2.0, ‘12': 1.0, ‘-15': 1.0, ‘-21': 1.0}
g4/C9 + g5/C12a	g5/C12a-F	46	14	None	50	0.99	0.101	0.405	ACAGCCGCCTAGC ATTTCATCACG	61	61	{‘0': 53.0, ‘1': 1.0, ‘2': 1.0, ‘-1': 4.0, ‘-2': 1.0, ‘-3': 30.0, ‘-5': 1.0, ‘-8': 2.0, ‘10': 1.0, ‘11': 1.0, ‘12': 2.0, ‘-21': 2.0}

Definitions of parameters identified by ICE analysis were provided by Synthego as follows: (1) ICE: indel percentage (all edit events, regardless of whether they are expected to introduce a KO); (2) KO-Score: all edits expected to introduce a KO (frameshift mutations or fragment deletions of 21 bp or greater); (3) ICE-d: reflects the discordance between the edited and control sequencing traces. Specifically, the ICE-d reflects the **Δ**d, or the difference between the control and edited alignment before the cut site and after the cut site. The ICE-d score typically lines up with the indel score; (4) mean discord before/after: mean discordance score before or after the first cut site, ranging from 0 to 1 (a minimum score of 0 indicates no discordance between the control and edited sequences, a maximum score of 1 indicates high discordance); (5) guide sequence: guide sequences input into the ICE tool; (6) control or edit sample quality score: median Phred score (0–62) of the region before the first cut site in the control or edited sample Sanger file, excluding initial noisy positions; and (7) indels: indels predicted to be present in the edited sample and the relative contribution of each indel (“indel size”: contribution percentage).

Additionally, we sequenced the J1.1 T cells with NF4 combination treatment after PCR amplification of the DNA fragment containing the g5/C12a target site (HIV ref/3′-LTR) using a g5-specific forward sequencing primer (Table 6). Because g5/C12a-mediated HIV gene editing generates staggered-end DSB, the treatment-induced indel and KO mutations are different from the g4/C9-generated blunt-end DSB. As shown in Figure 6C, the control (gc/C9 plus gc/C12a)-treated cells exhibited 100% conservation in the DNA sequence compared to the untreated cells, while the g5/C12a and g4/C9 plus g5/C12a-treated cells had significant DNA cleavage and indel mutations, with a high level of noise at and beyond the g5/C12a target site (rectangle framed). In addition, ICE analysis revealed higher indel and KO mutations (44%–46% and 14%–17%, respectively) for g5/C12a and g4/C9 plus g5/C12a RNP-treated cells (Figure 6D). Details of the ICE analysis are shown in Table 7.

Together, these sequencing data confirm HIV DNA cleavage with remarkable indel and KO frequencies at the target genes in the g4/C9 and/or g5/C12a RNP-treated cells, providing genomic evidence of HIV disruption by this treatment.

### Assessing potential cytotoxicity of the combination treatment with g4/C9 plus g5/C12a

Lactate dehydrogenase (LDH) is a cellular enzyme that is quickly released into the culture medium upon cell damage and is widely used for quantitative measurement of cellular cytotoxicity. We assessed LDH release from J1.1 T cells with NF4 treatment. We did not observe any significant differences in LDH release in the presence or absence of PMA stimulation, indicating no cytotoxic effects resulting from the treatments (Figure S1D). We also determined the percentage (%) of cell viability by MTT assay in J1.1 T cells with NF4 treatment. As shown in Figure S1E, we did not observe any significant changes in cell viability among the treatment groups compared to the untreated (no nucleofection) or control-treated cells, suggesting that the treatment with g4/C9 plus g5/C12a does not cause cell cytotoxicity.

## Discussion

Disrupting the HIV genome in infected cells via gene editing is a promising approach to the HIV cure. Gene editing using plasmid- or viral-based CRISPR technology in human applications has safety concerns and potential adverse effects, as it can be a “double-edged” sword in clinical settings.^47^ In this study, we used synthetic gRNAs for Cas9 and Cas12a RNPs as a non-viral formulation to inhibit HIV replication via disrupting the HIV genome in latently infected T cells. We demonstrated that gRNA/Cas9 and gRNA/Cas12a RNPs can efficiently suppress viral replication and expression in HIV-infected cells without causing significant cytotoxicity. According to our previous study, gRNA4/Cas9 demonstrated the most effective HIV suppression by targeting the vpr/tat region.^22^ In the current study, gRNA5/Cas12a exhibited the most effective HIV suppression by targeting the 3′-LTR/Nef region, possibly due to the fact that LTR is a repeat sequence that is identical at both ends of the provirus, suggesting that gRNA5/Cas12a likely has two different cut sites in the provirus, leading to possible removal of most of the proviral DNA. Given their different mechanisms of action, target sites, and cleavage ends, sequential treatment with gRNA4/Cas9 followed by gRNA5/Cas12a RNPs, and vice versa, and repeated treatments with gRNA4/Cas9 plus gRNA5/Cas12a in combination without or with ART (RAL plus DRV) led to an almost complete cessation of HIV activities. DNA sequencing showed that these gene editing RNPs can precisely cleave the integrated HIV DNA, resulting in a significant reduction in viral replication and expression. These findings highlight the potential of our synthetic gRNA4/Cas9 and gRNA5/Cas12a RNPs as promising therapeutic regimens for eradicating HIV infection. A working model of HIV gene editing by synthetic gRNA4/Cas9 plus gRNA5/Cas12a RNPs is shown in the Graphical abstract, depicting the mechanisms by which these gene editing therapeutics can induce HIV DNA damage and repair, leading to HIV gene disruption and viral eradication.

Conventional CRISPR expression methods, using plasmid DNA, mRNA, or viral vectors, often exhibit inconsistent transcriptional/translational activity and prolonged, uncontrolled gene expression *in vivo*, which can increase the risk of off-target effects and unwanted immune reactions.^48^ The synthetic gRNA/Cas RNP technology used in our study offers a direct and transient gene editing method that is more controllable and safer compared with the traditional expression systems. Out of ten selected Cas12a-directing gRNAs (having the highest levels of conservation among different HIV strains and no predicted off-target overlap with the human genome), gRNA5/Cas12a (targeting the HIV *Nef/3′-LTR* genes) in combination with gRNA4/Cas9 (targeting the HIV *Vpr/Tat genes)*^22^ exhibited the most effective antiviral activity by disrupting HIV proviral DNA and thus reducing HIV early and late RT transcripts, and mRNA and protein expression. These findings suggest that these HIV genomic regions represent optimal targets for a combined gRNA/Cas9 and gRNA/Cas12a gene editing therapeutic regimen for HIV gene disruption and viral eradication.

Evaluation of indel rates and KO frequencies following CRISPR-mediated DNA damage and repair is important for confirming its on-target gene editing and cleavage. We performed Sanger DNA sequencing to confirm the precise cleavage of the HIV target genes and aligned the sequences to determine the indel and KO frequencies in RNP-treated cells. The presence of DNA damage repair-generated indel or KO induces variability in the target sequences, since NHEJ may repair each transfected cell differently following the CRISPR-mediated DNA cleavage. This variability complicates Sanger sequencing, as the resulting DNA population consists of a pool of mixed products that differ in indel and KO frequencies. Additionally, because it is difficult to achieve 100% nucleofection efficiency, the cell population used for DNA sequencing included both edited and unedited cells. As a result, the sequencing chromatogram is usually clear up to the cleavage site, but becomes “noisy” at (and beyond) the target site, reflecting the mixed gene editing/repair outcomes (see Figure 6). One workaround for this issue might be to use GFP-Cas9 and mCr-Cas12a labeled RNPs to sort or subclone the treated cells and perform next-generation sequencing, which is ongoing in our laboratory.

In this study, we observed some HIV rebound after a single-targeting treatment with either gRNA4/Cas9 or gRNA5/Cas12a RNPs. Possible reasons for this viral rebound could include: (1) selection pressure and clonal outgrowth, i.e., due to limited transfection efficiency, the untreated cells with active HIV infection may grow faster, outcompeting the treated cells undergoing HIV gene editing. As cells divide, new viral mRNA/protein may be produced in the untreated cells or from the unedited genes. Over time, these unaffected cells outgrow in culture, lowering the observed HIV gene editing efficiency. (2) Limited nucleofection efficiency does not guarantee delivery of the RNPs into every HIV-infected cell, and thus the untreated cells can produce new viral progeny, which can infect/reinfect the cells that are already treated/edited. (3) The gRNA/Cas complexes may be tapered off or degraded with cell divisions following this transient treatment. (4) Incomplete target gene editing and imperfect DNA repair via the NHEJ and/or HR pathways, resulting in partial restoration/recovery of the target gene. (5) Epigenetic silencing or activation, i.e., if HIV KD is mediated by a CRISPRi (dCas12a fused with KRAB or similar), the repressive mark gradually diminishes over time, leading to HIV rebound. These possibilities may cause viral rebound following viral escape, leading to viral spread and reinfection. Thus, treatment with gene editing RNPs along with ART, as performed in our study, can improve the overall antiviral efficacy.

Although we did not observe any significant cytotoxic effects in J1.1 T cells from the repeated treatments with gRNA4/Cas9 and gRNA5/Cas12a RNPs, off-target effects are always a concern due to target MM by CRISPR-mediated gene editing. Theoretically, any unexpected off-target effects could cause structural chromosomal rearrangement in the host cell, potentially resulting in oncogene activation or genome instability,^49^^,^^50^^,^^51^ which warrants investigation into its potential off-target effects before its clinical application. Further analysis of off-target effects could benefit from the use of cutting-edge techniques,^52^ such as anchored primer enrichment (GUIDE-seq), *in situ* detection (BLISS), *in vitro* selection libraries (CIRCLE-seq), chromatin immunoprecipitation (ChIP), DISCOVER-Seq, translocation sequencing (LAM PCR HTGTS), and *in vitro* genomic DNA digestion (Digenome-seq and SITE-Seq).

### Limitations of the study

This study has several limitations, including the lack of evaluation of the long-term inhibitory effects of gRNA4/Cas9 and gRNA5/Cas12a RNPs and the potential of *in vivo* immune responses. Previous studies have shown that virus escape can occur when using a single gRNA, thus reducing the efficiency of HIV elimination,^27^^,^^28^^,^^29^^,^^30^^,^^31^ whereas a combination of gRNAs could improve viral suppression and clearance.^15^^,^^22^^,^^31^^,^^32^ In this study, we showed that repeated treatments with gRNA4/Cas9 and gRNA5/Cas12a in combination almost completely abolished HIV activities. Using a synthetic, transient RNP formulation is a fairly unique approach in HIV curative research. Unlike stable or long-term expression systems, which may potentially lead to oncogenesis, off-targeting, and unwanted immune responses, a transient RNP is not expected to affect the new generations of replicating cells because it will be degraded over time and thus is considered a safer and more controlled approach for genome editing. These factors should be taken into consideration when formulating future antivirals for clinical application. In our preliminary studies, we compared the efficiency of transfection versus nucleofection *in vitro*, and we chose the nucleofection delivery for our gRNA/Cas RNPs in this study. Clearly, this is not a suitable delivery method for clinical application. We are working on other clinically relevant strategies, using engineered LNPs or exosomes to deliver our selected gRNA/Cas RNPs to CD4^+^ T cells or monocytes *in vivo*. We anticipate that the development of alternative LNP- or exosome-based delivery systems could make the application of synthetic gRNA/Cas RNPs feasible in combination with ART to achieve a durable and functional HIV cure.

In summary, this study provides proof-of-concept that synthetic gRNA4/Cas9 and gRNA5/Cas12a RNPs can efficiently disrupt HIV replication and expression in latently infected T cells. Further development of multiplexed gRNA4/Cas9 and gRNA5/Cas12a RNPs, using engineered LNPs or exosomes for their delivery, may facilitate their clinical applications.

## Resource availability

### Lead contact

Requests for further information and resources should be directed to and will be fulfilled by the lead contact, Zhi Q. Yao (yao@etsu.edu).

### Materials availability

This study did not generate new unique physical materials or reagents.

### Data and code availability

• Data will be made available upon request.
• This study does not report original code. All analyses were performed using GraphPad Prism software and publicly available standard software as detailed in the STAR Methods.
• Any additional information required to reanalyze the data reported in this article will be available from the lead contact upon request.

## Acknowledgments

This work was supported by National Institutes of Health (United States) grants R21AI179794, R21AI157909, R01AI177624; VA Merit Review Awards 1I01BX006270, and VA-TTP BRAVE award OP2023-01943 (to Z.Q.Y). This publication is the result of work supported with resources and the use of facilities at the James H. Quillen Veterans Affairs Medical Center. The image for Graphical abstract has been prepared by BioRender. The contents in this publication do not represent the views of the Department of Veterans Affairs or the United States Government. VA: Veteran affairs.

## Author contributions

Conceptualization, funding acquisition, project administration, and supervision, Z.Q.Y.; data curation, formal analysis, methodology, and investigation, P.B., L.W., M.S, and J.S.P; resources, validation, software, and visualization, A.C.H., Y.Z., J.Z., H.K.O., J.W.L., and X.Y.W.; writing – original draft, Z.Q.Y. and P.B.; writing – review and editing, S.N., M.E.G., J.P.M., and Z.Q.Y.

## Declaration of interests

The authors declare no competing financial interests.

## STAR★Methods

### Key resources table

**Table:** 

REAGENT or RESOURCE	SOURCE	IDENTIFIER
**Antibodies**		

HIV-1 Core antigen-RD1, KC57	Beckman Coulter	Cat# 6604667; RRID: AB_1575989
HEP-20068 Monoclonal anti-HIV-1 p24 Gag protein, clone AG3.0	NIH HIV Reagent Program	BEI Resources Repository, Cat# HRP-20068; RRID: AB_3741631
β-Actin (8H10D10) Mouse mAb (HRP Conjugate)	Cell Signaling Technology	Cat# 12262S; RRID: AB_3741633
Anti-mouse IgG, HRP-linked Antibody	Cell Signaling Technology	Cat# 7076P2; RRID: AB_3741634

**Bacterial and virus strains**		

Human Immunodeficiency Virus 1 (HIV-1) Lymphadenopathy-Associated Virus (LAV)-Infected Jurkat E6 Cells (J1.1)	NIH AIDS reagent program	AF324493.2

**Biological samples**		

Primary CD4 T cells	CIIDI Biorepository	HIV-23-79, HIV-23-113, HIV-22-233, HIV-22-282, HIV-22-341, HIV-23-191, HIV-23-150, HIV-23-168

**Chemicals, peptides, and recombinant proteins**		

Corning™ RPMI 1640 Medium (Mod.) 1X with L-Glutamine	FisherScientific	MT10041CV
Gibco™ Penicillin-Streptomycin (5,000 U/mL)	Fisher Scientific	15-070-063
Gibco FBS, HI	FisherScientific	A5256801
L-glutamine	Fisher Scientific	25030081
DEPC Treated water	ThermoFisher	AM9915G
Phorbol 12-myristate 13-acetate	Millipore Sigma	16561-29-8
SE cell line 4D-Nucleofector™ X Kit L	Lonza	V4XC-1024
Cas9-NLS purified protein	QB3 Macrolab, UCB	N/A
Edit-R crRNA Non-targeting Control #1, 20 nmol	Horizon discovery	U-007501-01-20
SpCas9-gRNA4, 5 nmol	Millipore Sigma	N/A
Edit-R tracrRNA	Horizon Discovery	U-002005-200
Alt-R® Cpf1 Electroporation Enhancer, 10 nmol	IDT	467331107
Alt-R™ L.b. Cas12a (Cpf1) Ultra, 500 μg	IDT	10007923
Alt-R™ L.b. Cas12a crRNAs, 10 nmol	IDT	N/A
IDTE pH 7.5 (1X TE Solution)	IDT	11-05-01-05
Invitrogen eBioscience™ Fixable Viability Dye eFluor™ 450	Fisher Scientific	65-0863-14
iTaq™ Universal SYBR® Green Supermix	Bio-Rad	1725124
SE cell line 4D-Nucleofector™ X Kit L	Lonza	V4XC-1024
High-Capacity cDNA Reverse Transcription Kit	Thermo fisher	4368814
RNase Inhibitor	Thermo fisher	N8080119
PureLink™ Genomic DNA Mini Kit	ThermoFisher	K182001
QIAquick Gel Extraction Kit (50)	Qiagen	28704
Cell Proliferation Kit I (MTT)	Millipore sigma	11465007001
Cytotoxicity Detection Kit (LDH)	Millipore Sigma	11644793001
Gibco™ DPBS, no calcium, no magnesium	FisherScientific	14-190-250
HIV1 p24 ELISA Kit	abcam	ab218268
QIAquick Gel Extraction Kit (50)	Qiagen	28704
Darunavir	NIH AIDS Reagent Program	ARP-11447
Raltegravir	NIH AIDS Reagent Program	HRP-11680
RIPA buffer	Boston BioProducts	BP-407
Thermo Scientific™ Pierce™ BCA Protein Assay Kit	Thermo scientific	PI23227
Amersham ECL Prime Western Blotting Detection Reagent	Cytiva Lifesciences	RPN2236
TBS, Tris-Buffered Saline, 10X Solution, pH 7.4, 6X1L	Fisher Scientific	BP24711
Thermo Scientific Restore Western Blot Stripping Buffer	Fisher Scientific	PI21059

**Critical commercial assays**		

DNA sequencing	Azenta genewiz	N/A

**Experimental models: Cell lines**		

Jurkat J1.1 T cells	NIH AIDS Reagent Program	N/A
ACH-2	NIH AIDS Reagent Program	N/A
SupT1	NIH AIDS Reagent Program	N/A

**Oligonucleotides**		

gRNAs specific for Cas12a	See Table 1 from Table section	N/A
Primers and probe used for ddPCR	See Table 5 from Table section	N/A
Primers used for qPCR and HIV-1 DNA sequencing	See Table 6 from Table section	N/A

**Software and algorithms**		

ImageLab	Bio-Rad	N/A
CHOP-CHOP program	Online	N/A
Clustal Omega multiple sequence alignment tool	EMBL-EBI	N/A
ICE CRISPR analysis tool	Synthego	N/A
BioRender	BioRender	N/A
CasOFFinder	CRISPR RGEN Tools	N/A
GraphPad Prism v10	GrapgPad software	N/A
FlowJo V10	Tree Star	N/A
Chromas Tool	Technelysium DNA sequencing software	N/A
QX Manager software	Bio-Rad	N/A
ClustalW	Online	N/A

### Experimental model and study participant details

#### HIV-infected cell culture and reactivation

Latently HIV-infected J1.1 T cells and ACH-2 T cells (obtained from the NIH AIDS Reagent Program) were cultured in complete RPMI 1640 medium, supplemented with 10% heat-inactivated FBS (Fisher Scientific, Waltham, MA; Cat # A5256801), 100 IU/mL penicillin-streptomycin (Fisher Scientific; Cat # 15070063), and 2 mM L-glutamine (Fisher Scientific; Cat# 25030081) at 37°C in a 5% CO_2_ incubator. For HIV reactivation and latency reversal, confluent cells were stimulated with 20 ng/mL Phorbol 12-myristate 13-acetate (PMA) (Millipore Sigma; Burlington, MA; Cat # 16561298) for 2 h, based on optimal conditions established in our previous study.^22^ Following stimulation, the cells were washed twice to remove PMA, incubated for an additional 24 h to allow latency reversal, and subsequently harvested for the assessment of mRNA replication and protein expression.

#### Primary CD4 T cells from PLWH on ART

Peripheral blood mononuclear cells (PBMCs) were isolated from the whole blood of 8 PLWH using Ficoll-Paque solution (GE Healthcare; Piscataway, NJ) and density gradient centrifugation separation. CD4 T cells were purified from PBMCs using a CD4^+^ T cell negative selection kit (Miltenyi Biotec; Auburn, CA). The human PBMCs employed in this study were from deidentified samples obtained from an existing Center of Excellence in Inflammation, Infectious Diseases and Immunity Biorepository, approved by the ETSU/VA IRB and managed via the BioStore III system. Informed consent was obtained from all participants, and this study was approved by the ETSU institutional biosafety and chemical safety committee. We chose PLWH who were viremic due to 1) being acutely infected and newly starting ART or 2) being chronically infected but ART was interrupted and just resumed, thus HIV RNAs were detectable at the time of blood collection. Given their low CD4 counts, the purified CD4 T cells were combined to ensure sufficient cell numbers for the ddPCR assay. The demographic features of PLWH used in this study are shown in Table 4.

#### Ethics oversight

The protocol of this study was approved by the joint Institutional Review Board (IRB) of East Tennessee State University and James H. Quillen VA Medical Center (ETSU/VA IRB# 0519.24s) and written informed consent was obtained from all participants.

### Method details

#### Design of gRNA/Cas RNPs

To design virus-specific gRNAs/Cas12a, the HIV pNL4-3 strain (∼9.8 kb complete sequence, PubMed Accession No. AF324493.2) was mapped using the NCBI nucleotide BLAST tool, focusing on two long terminal repeats (5′-LTR, 3′-LTR) and 9 protein-coding genes. A total of 10 gRNAs for Cas12a were designed using the online CHOP-CHOP program,^38^ considering parameters such as genomic location, strand orientation, GC content, self-complementarity, efficiency score, and off-target effects. The selection criteria prioritized higher efficiency, no off-target hits, GC content % > 40 for stability, and no self-complementarity.^49^^,^^50^^,^^51^ The virus-specific gRNAs designed to target one or multiple HIV genes and the non-targeting scramble gRNA control (gc) were synthesized by Integrated DNA Technologies (IDT, Coralville, IA) with RNA-stabilizing modifications (5′ and 3′ Alt-R). The Recombinant L*.b*. ND2006-derived Cas12a (purchased from IDT) was purified from an *E. coli* strain expressing Cas12a with proprietary mutations to improve both on-target editing and temperature tolerance. The Cas12a contains a nuclear localization sequence (NLS) to guide the gRNA/Cas12a RNP complex to the nucleus and a C-terminal 6-His tag for protein purification. The Cas9 protein from *staphylococcus pyogenes* was provided by the QB3 Macro Laboratory at the University of California, Berkeley. The Cas9-directing gRNA4 was purchased from Millipore Sigma. The tracrRNA and non-targeting gRNAc were purchased from Horizon Discovery (Waterbeach, UK), as described in our previous study.^22^ The synthesized gRNAs and Cas9 or Cas12a proteins were stored in −80°C refrigerator and remained functionally stable for at least 6 months.

#### Preparation of gRNA/Cas RNPs for nucleofection

The gRNA/Cas9 RNP and gRNA/Cas12a RNP complexes were freshly prepared before each experiment using the manufacturer’s protocol with minor modifications. To make the gRNA/Cas9 RNP complex, 160 μM of gRNA was mixed with 160 μM tracrRNA and incubated at 37°C for 30 min, and then 80 μM of this RNA duplex was mixed and incubated with 40 μM of Cas9 protein at 37°C for 15 min. To make the gRNA/Cas12a complex, 75 μM of gRNA was incubated with 67 μM of Cas12a protein at room temperature for 20 min. Before nucleofection, Alt-R Cas12a (Cpf1) electroporation enhancer (IDT, Cat # 1076300) was used according to the manufacturer’s instructions. For the combination treatment with the two RNP complexes, the assembled gRNA/Cas9 RNP and gRNA/Cas12a RNP complexes were mixed at a 1:1 ratio (with the amount of each RNP complex in the mixture equaling half of the total amount of each RNP complex when used separately) before cell nucleofection for J1.1 T cells, while a full dose of each RNP at 1:1 ratio was used before cell nucleofection for ACH-2 cells.

For both single and combination treatments, 2x10^6^ J1.1 or ACH-2 T cells were nucleofected with the gRNA/Cas9 and/or gRNA/Cas12a RNPs using the SE cell line 4D-Nucleofector X Kit L (Lonza, Basel, Switzerland; Cat #V4XC-1024) in a 4D nucleofector core unit with the specific program for E6-1 T cells. Forty-eight hours post-nucleofection, the cells were stimulated with PMA for 2 h, washed twice, and cultured for an additional 24 h, followed by flow cytometry, RT-qPCR, WB, and ELISA assays for viral assessment. Notably, the treated cells were grown until enough expansion for each treatment with 2x10^6^ cells and for repeated treatments, a portion of the transfected cells was maintained through multiple passages or cryo-preserved in freezing medium (40% RPMI, 50% FBS, 10% DMSO) in liquid nitrogen for later use. To determine the role of HIV spread in viral rebound after treatment and reinduction during cell culture, we cultured the gRNA/Cas-treated cells in the presence or absence of antivirals with raltegravir (100 nM) and darunavir (1 μM, both from HIV reagent program), followed by virology assays as described in the Results.

#### Flow cytometry

To determine the levels of intracellular HIV p24 antigen expression in the treated cells, a two-step immunostaining procedure was performed following cell nucleofection, HIV reactivation, and latency reversal. For live cell staining, cells were washed with DPBS and stained with Invitrogen eBioscience Fixable Viability Dye eFluor 450 (Fisher Scientific, Cat # 65-0863-14) according to the manufacturer’s protocol. The cells were permeabilized using the Fixation/Permeabilization reagents (Fisher Scientific, Cat # 50-112-9082) and then stained with an RD1-conjugated anti-HIV core (p24) monoclonal antibody (Beckman Coulter, Brea, CA; Clone KC57, Cat # 6604667). The cells were analyzed by BD FACSymphony A3 flow cytometer (BD Bioscience, San Jose, CA) and FlowJo v10 software (Tree Star, Ashland, OR). Unstained and single-stained cells were used for the gating strategy and multicolor compensation. Figure S1C shows the gating strategy used for cell analysis.

#### Western blot

Western Blot was performed to measure the expression of HIV p24 protein on the 4th time combinational RNP-treated J1.1 cells. The whole-cell lysates were prepared using RIPA buffer (Boston BioProducts, Ashland, MA) in the presence of protease inhibitors (Thermo Scientific, Rockford, IL). Protein concentration was measured by Pierce bicinchoninic acid (BCA) protein assay kit (Thermo Scientific). Protein extracts were separated by SDS-PAGE, transferred to polyvinylidene difluoride membranes, and pre-blocked with 5% nonfat milk in 0.1% Tween 20 and Tris-buffered saline (TBS). The membranes were incubated with the anti-p24 primary antibody (NIH AIDS Reagent Program) or anti-β-actin antibody (Cell Signaling Technology, Danvers, MA) overnight. After washing, the membranes were incubated with horseradish peroxide-conjugated secondary antibodies (Cell Signaling Technology) for 2 h at room temperature. Protein bands were detected using Amersham ECL Prime Western blotting detection reagent (Cytiva Lifesciences). The bands were captured and quantified using the ChemiDoc MP imaging system (Bio-Rad, Hercules, CA).

#### RT-qPCR

Total RNA was extracted from 1x10^6^ treated cells using the Qiagen RNeasy Mini Kit (QIAGEN; Cat # 74106). The RNA concentration was determined by a BioTek Synergy H1 microplate reader, and cDNA was synthesized from 150 ng RNA using the High-Capacity reverse Transcription Kit (Applied Biosystems, Waltham, MA; Cat # 4368813). 1 μL cDNA was used for RT-qPCR to amplify HIV mRNA with the iTaq Universal SYBR green master mix (Bio-Rad, Hercules, CA; Cat # 1725124). The PCR primers to amplify HIV and control mRNAs used in this study are listed in Table 6. The reverse primer specifically hybridizes with the poly(A) tail of the spliced HIV mRNA to avoid interactions with any aberrant RNAs and/or integrated or unintegrated proviral DNA. The PCR cycling conditions were as follows: one cycle of enzyme activation at 95 °C for 10 min, and then 35 cycles of denaturing at 95 °C for 15 s, annealing at 60 °C for 15 s, and extension at 72 °C for 15 s. Gene expression levels were determined by the 2^−ΔΔCt^ threshold method, normalized first to GAPDH levels and then to the non-targeting gc group, and are presented as fold change. To detect the integrated proviral DNA, genomic DNA was isolated and subjected to 30 cycles of pre-amplification with Alu-gag, gag only, or Alu only, followed by the 2nd qPCR amplification of R-U5, using the pre-amplified PCR products as the template with a method for detection of the integrated proviral DNA as described previously.^42^ The Alu-gag PCR method quantifies integrated HIV proviral DNA by pairing a host Alu repeat primer with an HIV gag-specific primer, enabling selective amplification of chromosomally integrated viral genomes while excluding unintegrated HIV DNA. Detection of HIV reverse transcription (RT) products, including HIV early R-U5 and late LTR-gag RT products, was performed as previously described.^43^ Reactions were performed in triplicates using Bio-Rad SYBR Green, and the relative levels of R-U5, LTR-gag, and Alu-gag gene products were normalized to β-globin.

#### Droplet digital PCR (ddPCR)

ddPCR was performed with a modified protocol as described previously,^53^^,^^54^^,^^55^ using the QX200 Droplet Digital PCR System (Bio-Rad, Hercules, CA). For HIV un-spliced RNA (usRNA) and multi-spliced RNA (msRNA) assays, 200 ng of cDNA was used per reaction, and 20 ng of cDNA was used for the RPP30 assay. Each 20 μL ddPCR reaction mix contained cDNA, 500 nM of primers, 250 nM of probe, and 2x ddPCR Supermix for Probes (Bio-Rad). The 20 μL reaction mix was loaded into DG8 cartridge with 70 μL of droplet generation oil (Bio-Rad), and droplets were generated using the QX200 Droplet Generator (Bio-Rad). 40 μL of droplets were transferred to a 96-well plate, and PCR was performed in a C1000 Touch Thermal Cycler (Bio-Rad) with the following cycling conditions: initial denaturation at 95 °C for 10 min; 40 cycles of 94 °C for 30 s and 60 °C for 60 s; and a final enzyme deactivation at 98 °C for 10 min, with a ramp rate of 2°C/s. Droplets were read using the QX200 Droplet Reader (Bio-Rad) with Direct Quantification of FAM fluorescence, and data were analyzed using QX Manager software (Bio-Rad). No-template controls (NTCs) and uninfected healthy subject (HS) controls were included in all assays to assess background and false-positive events. Primer and probe sequences used in this study are listed in Table 5.

#### HIV DNA sequence analysis

Following the 4th round of combination treatment, genomic DNA was isolated from J1.1 cells using PureLink Genomic DNA Mini Kit (Cat# K182001, Invitrogen). The DNA fragments (280–290 bp) were amplified by PCR using primers (Table 6) flanking the g4/C9 and g5/C12a cleavage sites (vpr/tat and nef/3′LTR, respectively). The PCR products were purified using the DNA Clean & Concentrator Kit (Zymo Research, Cat# D4004) and confirmed using 1% agarose gel electrophoresis. The extracted DNA fragments were sequenced by Sanger sequencing with appropriate forward and reverse primers (Table 6) at Azenta Genewiz (La Jolla, CA). The sequencing readouts were aligned and compared to the untreated and gc-treated cells. The Chromas tool (Technelysium DNA sequencing software) was used to read the nucleotide peaks, and the Clustal Omega multiple sequence alignment tool from EMBL-EBI was used to align and compare the nucleotide sequences of our PCR products. Synthego’s ICE tool was used to calculate the indel rates and KO frequencies of g4/C9 and/or g5/C12a-treated cells compared to the untreated and control-treated cells (Table 7).

### Quantification and statistical analysis

All data were analyzed using the GraphPad Prism V10 software (GraphPad Software, San Diego, CA) and are shown as mean ± SEM. The comparison between two groups was analyzed by a paired *t* test, and comparisons among multiple groups were carried out using a parametric one-way ANOVA or a non-parametric Friedman one-way ANOVA test, based on the normality results. *p* values <0.05 were considered statistically significant, and *p* values <0.01 were considered very significant.
