# Supplementary material for: Combination treatment with synthetic gRNA/Cas12a and gRNA/Cas9 ribonucleoproteins disrupts HIV replication and expression
Source: iScience. 2026 Apr 3;29(5):115606. doi: 10.1016/j.isci.2026.115606 (PMC13122203; doi:10.1016/j.isci.2026.115606)
Supplement: Document S1. Figure S1 and Methods S1 [file mmc1.pdf]

iScience, Volume *29*

Supplemental information

Combination treatment with synthetic gRNA/Cas12a and gRNA/Cas9 ribonucleoproteins disrupts HIV replication and expression

Puja Banik, Ling Wang, Madison Schank, Jaeden S. Pyburn, Addison C. Hill, Yi Zhang, Juan Zhao, Holly K. Orfield, Tabitha O. Leshaodo, Janet W. Lightner, Xiao Y. Wu, Shunbin Ning, Mohamed El Gazzar, Jonathan P. Moorman, and Zhi Q. Yao

Supplemental Figure 1

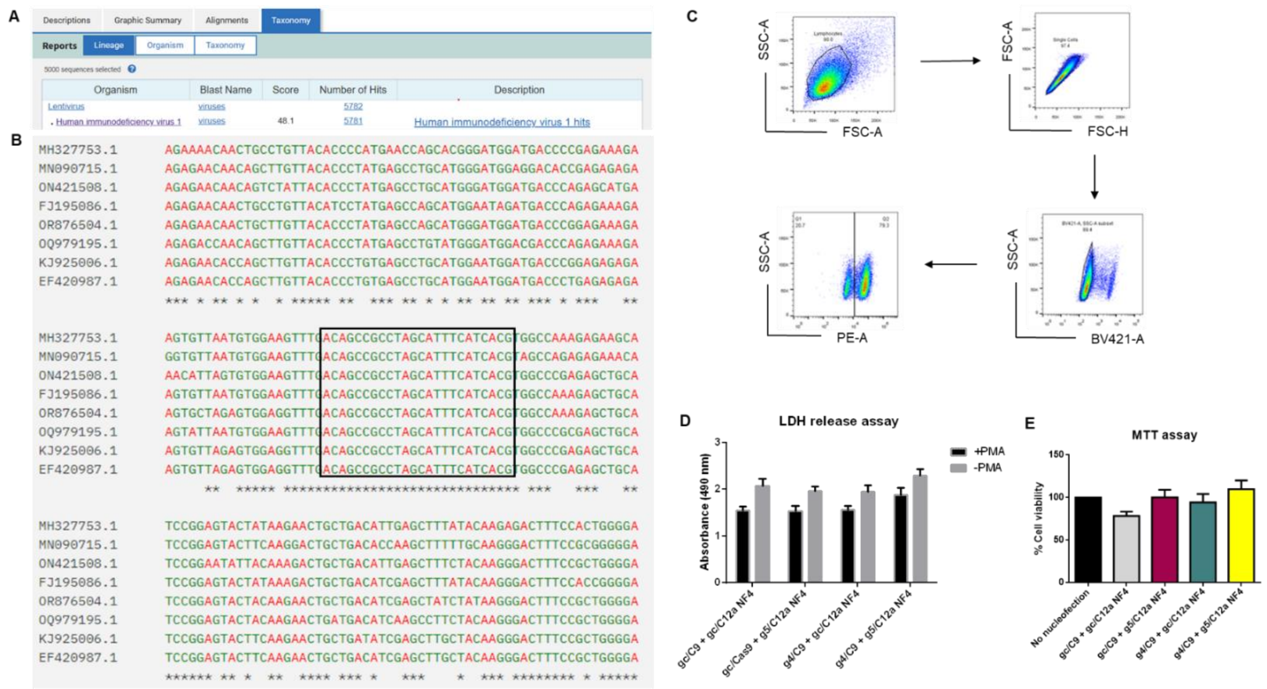

Figure S1. Sequence alignment of g5/C12a across major HIV strains and cytotoxicity assays of J1.1 cells with the treatment. A) NCBI nucleotide blast revealed a total of 5781 hits with the g5/C12a sequence across the published HIV partial or complete genome sequences. **B)** The g5/C12a sequence (rectangle framed) is located in a very conserved area across major HIV strains derived from different countries when analyzed by the Clustal Omega multiple sequence alignment tool. The * marked underneath the alignment represents identical nucleotides across the HIV strains. **C)** Representative pseudocolor plots for flow cytometry gating strategy are shown. Briefly, viable 450^- cells were gated after gating scatter and singlets. Next, the expression of p24 was analyzed in viable cells. **D-E)** LDH release and MTT assays of J1.1 cells with NF4 treatment by gc/C9 + gc/C12a, gc/C9 + g5/C12a, g4/C9 + gc/C12a, and g4/C9 + g5/C12a RNPs, respectively.

Methods S1

LDH assay. LDH release was measured using a commercial kit (Roche, Basel, Switzerland; Cat # 11644793001). Briefly, after the 4th combination treatment, J1.1 cells were cultured in RPMI

1640 medium with or without PMA stimulation for 2 h, washed twice, and cultured for an additional 24 h. Approximately 100 µl of supernatant was incubated with 100 µl of freshly prepared reaction mixture at room temperature (RT) for 30 minutes according to the instructions by the manufacturer. The LDH release absorbance was measured at 490 nm with a BioTek Synergy H1 microplate reader.

MTT assay. MTT assay was performed using a commercial kit (Millipore Sigma; Cat # 465007001). Briefly, after the 4th combination treatment, J1.1 cells were cultured in cRPMI 1640 culture medium with PMA stimulation for 2 h, washed twice, and cultured for an additional 24 h at a 20000 cells/100 µl cRPMI ratio. The next day, 10 µl of MTT labeling reagent was added in each well at a final concentration of 0.5 mg/ml for 4 h at 37 ºC, followed by the addition of 100 µl of solubilization buffer and incubation overnight according to the manufacturer’s instructions. The MTT absorbance was measured at 600 nm using a BioTek Synergy H1 microplate reader.
